# Evaluation of paraoxonase I and hemoglobin levels in farmers and agricultural workers in relation to organophosphorus and carbamate levels in their blood and urine samples: A cross sectional study

**DOI:** 10.12688/f1000research.131690.2

**Published:** 2023-10-16

**Authors:** Vinutha Bhat, Pragati Nayak, Shankar Bakkannavar, Padmanabha Udupa

**Affiliations:** 1 Poison Information Center, Department of Forensic Medicine and Toxicology, Kasturba Medical College, Manipal, Manipal Academy of Higher Education (MAHE), Manipal, 576104, India; 2 Biochemistry, Kasturba Medical College, Manipal, Manipal Academy of Higher Education (MAHE), Manipal, Karnataka, 576104, India; 3 Forensic Medicine and Toxicology, Kasturba Medical College, Manipal, Manipal Academy of Higher Education (MAHE), Manipal, Karnataka, 576104, India

**Keywords:** Pesticides, toxicity, organophosphorous poisoning, carbamate poisoning, paraoxanase I (PONI), haemoglobin

## Abstract

**Background:** Pesticides are chemicals that have become common household products in developing countries. The purpose of pesticides is to manage agricultural work. The majority of pesticides for indoor and agricultural use are carbamate and organophosphorus. Toxicity is caused due to excess and improper use or disposal of these chemical agents. Slow exposure to pesticides causes chronic poisoning whereas rapid exposure causes acute poisoning. The paraoxonase I (PON 1) enzyme has a role in detoxifying some of the oxon derivatives which thereby inhibit acetylcholinesterase and butyrylcholinesterase.

**Methods:** This study analyzed farmers who were exposed intermittently to organophosphorus and carbamates pesticides during farming for more than five years. Serum paraoxonase I was evaluated by colorimetry method, and hemoglobin levels were evaluated using portable Fresenius Kabi haemoglobinometer.

**Results:** The study showed that the pesticides were found in the blood and urine samples of farmers and there was an alteration of paraoxonase I and hemoglobin levels in them due to the exposure of pesticides in large quantities over some time. The present study showed around 81% of the participants who were intermittently exposed to pesticides for more than five years were detected with pesticide toxicity. The paraoxonase I level was altered in farmers who were positive for organophosphorus and carbamate pesticides. The hemoglobin level did not show much variation among the farmers exposed to pesticides. This may be due to the lifestyle of the subjects, climatic variations and also their eating habits.

**Conclusions:** The study suggested that there was alteration in the levels of PON1 and hemoglobin in farmers and agricultural workers with positive organophosphorus and carbamates in their blood and urine samples. As our study was done without quantifying the amount of pesticides, further studies can be done by quantifying the pesticide level and comparing it with the paraoxonase I level.

## Introduction

Pesticides are synthetic chemical compounds that are used to destroy pests including insects and unwanted plants. As a result of its easy availability, it has become a commonly used agricultural product in rural areas of developing countries. ^1^ Pesticide poisoning is a major challenging problem all over the world. ^2^ Exposure to pesticides, specially of OPs is an important toxicological issue of concern, in India. Acute pesticide poisoning is an important cause of morbidity and mortality in India. ^3^

OP and CBM pesticides are common household insecticides used commonly for agricultural purposes in developing countries. ^4^^,^^5^ In India, OP, CBM, organochlorine, and pyrethroids are the commonly used pesticides. ^6^ Most of these pesticides are sold directly from shops due to the lack of special rules and regulations regarding the use and sale of pesticides in countries like India. ^2^

Chronic and acute exposure is mainly through residue of pesticides sprayed to crops and vegetables. ^7^ According to a recent study, the various food commodities such as cereals and vegetables contain pesticide residues. ^8^ Hence the farmers and agricultural workers exposed to pesticides are at risk of acute or chronic poisoning.

PON I, an Esterase, can detoxify pesticide derivatives in human serum. ^9^ The primary function of the PON I enzyme is likely to be related to its antioxidant properties. ^10^ However, it plays an important role in the pesticide toxicity pathway by helping in hydrolyzing several oxon derivatives. ^11^ PON I activity of an individual can vary to a large extent. It is reported that PON I levels can vary by at least 13-fold and the activity up to 40-fold. ^7^ This implies that the ability of individuals to detoxify OP would depend on their PON I activity. Pesticide poisoning inhibits the PON I enzyme which in turn inhibits acetylcholinesterase in synapse and on red blood cell (RBC) membrane and butyrylcholinesterase in plasma. ^12^ Accumulation of cholinesterases causes central nervous system depression or seizures, and respiratory failure. ^13^

The PON-I has a role in prevention of oxidative stress and inflammation, the level of which is affected in poisoning cases. ^14^ The lifespan of red blood cells can be shortened through oxidative damage and toxic mechanisms resulting in anaemia. Hemoglobin is also subjected to oxidative damage resulting the formation of methemoglobin which is nonfunctioning resulting in tissue hypoxia. ^14^

The present study shows the pesticide toxicity in farmers and agricultural workers who were exposed to pesticides for more than 5 years. Exposure was confirmed by asking the participants about their use/contact with pesticides, and toxicity was determined via the paraoxonase I and hemoglobin levels measured.

## Methods

### Ethics statement

Ethical approval was obtained from Institutional Ethics Committee (Kasturba Hospital, Manipal, which is a part of Kasturba Medical College, Manipal; No: IEC143/2017 dated 15.02.2017) before starting the study. The detailed plan of the study was explained to the 100 farmers and agricultural workers through the Participant Information Sheet (PIS) and on willingness to participate in the study, written informed consent in a printed form was obtained from the study participants. After agreeing to participate in the study, the participants voluntarily signed the form. The consent form was pre-validated and approved from the ethics committee. The consent was obtained for both participation and for academic publication purposes.

### Study design

This cross-sectional prospective observational study was conducted between October 2017 to April 2018 in the poison information center, part of the Kasturba tertiary care teaching hospital in South India. Both male and female agricultural workers were included in the study belonging to the age group 25–75 years. 100 farmers and agricultural workers (50 males and 50 females) of the native district (Udupi district of Karnataka) were included in the study and the blood and urine samples were collected from them in the hospital by the postgraduate medical students (interns) and faculty members. These farmers and agricultural workers were part of the health checkup camp arranged for them in the native district. A total of 240 agricultural workers were part of health checkup camp. Out of them based on inclusion criteria i.e. those with minimum 5 years of work in the field and more than 25 years of age were included in the study. Those farmers and agricultural workers were identified based on their identity card and initial information collected. The effects of chronic toxicity can be seen in individuals in several months to years after the exposure. ^15^ Hence, only the farmers and agricultural workers who were involved in agriculture for a period of at least 5 years (and who confirmed their exposure to pesticides) were included in the study.

During the study, the antecubital fossa was cleaned with antiseptic solution and through venipuncture by puncturing medial cutaneous vein or cubital vein or brachial vein, 2 ml of the blood was collected. The urine was collected in the labelled plastic container of 20 ml capacity.

### Estimation of hemoglobin level

Haemoglobin levels of each participant was evaluated using portable Fresenius Kabi haemoglobinometer. It is essentially a photometer which allows measurement of color intensity of solutions. The disposable micro cuvette acts as reaction vessel in which measurements are made. The reagents necessary for both release of Hb from erythrocytes and conversion of Hb to a stable-colored product are present in dried form on the walls of the cuvette. A small sample (typically 10 μL) of venous blood to the micro cuvette is introduced and micro cuvette is introduced into the instrument. The instrument is factory precalibrated using HiCN standard, and absorbance of the test solution is automatically converted to ctHb. The result is displayed in less than a minute. The displayed results were recorded.

### Qualitative estimation of pesticides

A total of 2 ml of each extracted blood and urine sample were processed qualitatively by performing thin-layer chromatography (TLC) by running against organophosphorus (Cypermethrin, Thimet and Chlorpyrifos) and carbamate (Anuferon) standards.

To one portion of the sample, two portions of the diethyl ether was added and vortexed. The upper layer was collected and considered as the treated sample. The extraction process was repeated two to three times.

The thin layer silica sheets were cut according to the number of samples and the size of the chamber. Linea were cut 1 cm away from the top and bottom cut ends of TLC sheets. The line below was for the sample application and the line above was considered as the solvent front. Simultaneously the glass chamber with the solvent system consisting of cyclohexane:acetone:chloroform [70:25:5] in it, was kept for saturation (at least for 10–15 min).

Spotting the standards and treated sample was done using the capillary tubes. The capillary tube was placed vertically on the TLC sheet repeatedly so that the solution was applied in several portions with intermediate drying (blow with cold or hot air). The organophosphorus and carbamate standards were spotted 5-6 times to increase the concentration and the treated samples are spotted 16–18 times for the same reason at the base line drawn. Then the TLC plates were kept in the saturated TLC chamber carefully without disturbing the solvent and allowed the solvent to run till the solvent front.

The solvent ascends through the layer by capillary action and causes the substances to separate. Once the solvent reaches the solvent front (near the top line) the TLC plates are taken out and kept for drying. The dried TLC plates are carefully placed in the visualizing chamber (iodine chamber) for 5–10 min till brown spots are visible.

The plates were then taken out and the spots were marked immediately. Localization of a substance was done with parallel runs with reference substances. The value used for evaluation was R _f _value (retention factor). R _f_ values of samples were calculated, and comparison was done with the R _f_ value of the standards. R _f_ value was defined as follows: R _f_ = Distance from starting point to middle of spot/Distance from starting point to solvent front.

The farmers and agricultural workers were categorized depending on the presence or absence of pesticides in their blood or urine samples into following 4 groups; carbamate toxicity, organophosphorus toxicity, both carbamate and organophosphorus toxicity and no pesticide toxicity.

### Estimation of paraoxanase I level

250 μl of diluted serum was added in 2 ml of tris HCl buffer containing 0.5 ml of 1 mmol of CaCl _2_, 0.5 ml of 2.5% methanol, and 0.5 ml of p-nitrophenyl acetate. The absorbance of the solution is measured at 405 nm. The intensity of the color developed is directly proportional to the amount of Paraoxanase present in the serum.

The data obtained were tabulated and analyzed using Statistical Package for Social Sciences (SPSS) version 21. Data was expressed as Mean ± SD. The significance value was set to p > 0.05. Inter comparison of data between the groups was assessed by ANOVA (One Way Analysis of Variance).

## Results

100 samples were collected from 100 farmers and agricultural workers belonging to the age group of 18 to 75 years involving both males and females. All the samples were divided into four groups based on the presence of the type of pesticide as; Group 1: CBM which was detected in 60 farmers; Group 2: OP positive in 13 farmers; Group 3: Absence of pesticide which was seen in 19 participants and Group 4: Both OP and CBM, positive in 8 farmers, as mentioned in Table 1 and *Underlying data*. ^16^

**Table 1.** Number of participants in carbamate, organophosphorus, combination of both, and no pesticide groupings.

Carbamate positive (1)	Organophosphorus positive (2)	No pesticide (3)	Organophosphorus and carbamate positive (4)
60	13	19	8

The four groups were analyzed statistically but did not show any significant relation with a p-value (p < 0.005). PON I level was comparatively low in participants positive for CBM, OP, and both CBM and OP positive when compared with participants who were detected negative for pesticide ( Table 2).

**Table 2.** Mean ± SD between hemoglobin and paraoxanase I levels.

Parameters	Group	N	Mean	Std. Error
Haemoglobin levels	1	60	13.4933	.27158
	2	13	12.8615	.64714
	3	19	13.4058	.54318
	4	8	11.6750	.56339
	Total	100	13.2491	.21810
Paraoxanase I level	1	60	32.9862	1.67419
	2	13	29.2869	2.22575
	3	19	40.3605	4.76337
	4	8	35.5688	5.96569
	Total	100	34.1130	1.47660

1: Carbamate toxicity, 2: Organophosphorous toxicity, 3: No Toxicity, 4: Combination of Carbamate and Organophosphorous toxicity.

Hemoglobin level was considerably low in participants containing both CBM and OP positive cases.

When the levels of haemoglobin and PON I, were compared between the groups, the statistically significant results were not shown ( Table 3). F value was 1.856 for intergroup comparison for hemoglobin and 1.780 for PON I which were statistically unsignificant with p value. We had outliers in the study. As a result, the data of one participant was not included in the analysis of intergroup comparison as shown in Table 3. We are confining our study to only adjusted estimates since we have no regression analysis included in our study.

**Table 3.** F (test value) and Sig (significance value) of haemoglobin and paraoxanase I level.

		Sum of Squares	df	Mean Square	F	Sig.
HB level	Between groups	25.820	3	8.607	1.856	.142
	Within groups	445.104	96	4.637		
	Total	470.925	99			
Paraoxanase	Between groups	1137.522	3	379.174	1.780	.156
	Within groups	20447.995	96	213.000		
	Total	21585.517	99			

When the inter group comparison i.e. OP positive cases (group 2 and 4) done, the PON I level was raised which was statistically significant as shown in Table 4.

**Table 4.** F (test value) and Sig (significance value) of paraoxanase I level in OP positive groups.

	Group	N	Mean	Median	SD	SE	df	P
Paroxanase	2	13	29.3	29.3	0.976	0.271	19	<0.001
	4	8	35.6	35.5	1.16	0.411		

## Discussion

Pesticides poisoning is an important toxicological issue all over the world. In rural areas of developing countries like India, pesticidal poisoning causes a greater number of deaths than rest of other diseases or infections. ^17^ Lack of regulatory control to the use and distribution of pesticides is one of the major reasons for these poisoning cases. ^18^ Organophosphorus and carbamate toxicity are the most common cases seen in agriculturally rich areas. ^19^

Management of pesticide poisoning cases is a greater challenge in developing countries. Though there are specific management protocols available, ^20^^,^^21^ research is still being performed around the world in view of their early diagnosis and more effective management. A number of biomarkers and bedside diagnostic tools are being developed for easy detection of pesticide exposure. ^22^ The levels of biomarkers of pesticide exposures in human invasive samples such as blood and non-invasive samples such as urine have been studied. ^3^^,^^23^^,^^24^

PON I is a high-density lipoprotein-associated enzyme that plays a critical role in the metabolism and detoxification of Organophosphorus pesticides as shown in many studies. ^8^^,^^10^ In the present study, the PON I level is comparatively less in Organophosphorus poisoning than in the other poisoning groups. Similar observations were mentioned by Vilanova and Sogorb, 1999; Sogorb and Vilanova, 2002. ^25^^–^^27^ A low level of PON I enzyme will increase the susceptibility to pesticides causing respiratory and neurological problems. ^28^ The higher levels of PON I activity in the patients may have substantially better detoxifying capacity against OP compounds as shown in research done by Hettagowdanahally V Rahul *et al*. ^29^ However, previous researchers have shown that the organophosphorus compounds directly inactivate the PON I affecting neither their synthesis nor clearance of the enzyme. ^1^ However, PON level is a risk factor, not the effect. Its level and polymorphism is affecting the capacity of detoxication and so the level of OP in blood may be higher as they are hydrolyzed and eliminated at lower rate. Therefore, it should be considered that the level of PON I in the individual is affecting the level of OPs remaining in blood and the possibility of toxic effects.

When the difference in the levels of paraoxonase and hemoglobin was considered, the lower values were observed for OP and CBM positive workers, although it was not statically significant. Cholinesterase inhibition due to pesticide poisoning leads to cellular dysfunction leading to oxidative damage of the RBC membrane. This leads to a lower level of hemoglobin levels in pesticide poisoning cases. In these cases, there is no statistical significance in hemoglobin levels which may be due to environmental factors or due to the food habits of the patients.

In the present study, around 85% of the participants were evaluated positively to various pesticides like OP and CBM. This concludes that there is no proper management for the use and disposal of pesticides. The studies have shown that PON I levels to a larger extent and detoxification of pesticides majorly Organophosphorus would depend on Paraoxanase I. ^7^ In those people with low level of PON I have lower capacity of detoxication, and consequently, higher level may be detected in their blood.

### Limitations

The study had no statistical significance since it had certain limitations. The quantification of organophosphorus and carbamates was not done in our study. Only the samples were analysed qualitatively for presence of organophosphorus and carbamates. Hence the considerable proportionate of lowering of PON I level probably was not statistically significant.

## Conclusions

Our study suggests that around 85% of the participants who were exposed to pesticides for more than 5 years were detected with presence of OP and CBM. The PON I level was low in participants who were positive for presence of pesticide than in the participants who were normal. Further studies can be done by quantifying the pesticide level and comparing it with the PON I level.

Early identification followed by effective management in the initial stages increases the rate of survival among pesticide toxic patients.

## Data Availability

Figshare: Evaluation of paraoxonase I and hemoglobin levels in farmers and agricultural workers in relation to pesticide levels in their blood and urine samples: A cross sectional study. https://doi.org/10.6084/m9.figshare.22595113.v3. ^16^ This project contains the following underlying data:
- FARMERS LIST - ALL.xlsx. Data are available under the terms of the Creative Commons Zero “No rights reserved” data waiver (CC0 1.0 Public domain dedication).
