# Supplementary material for: Evaluation of paraoxonase I and hemoglobin levels in farmers and agricultural workers in relation to organophosphorus and carbamate levels in their blood and urine samples: A cross sectional study
Source: F1000Res. 2023 Oct 16;12:Chem Inf Sci-478. Originally published 2023 May 10. [Version 2] doi: 10.12688/f1000research.131690.2 (PMC10915361; doi:10.12688/f1000research.131690.2)
Supplement: Peer review history

# Reviewer report: Reviewer response for version 2

**DOI:** 10.5256/f1000research.156722.r251682

**Published:** 2024-03-05

**Authors:** Shruthi Prabhakar

**Affiliations:** 1 Department of Forensic Medicine, Saveetha University, Chennai, Tamil Nadu, India

Paraoxonase I is an enzyme that plays a role in detoxifying organophosphorus compounds. Hence it is an important determinant of OP toxicity. Evaluating its levels can provide information on the individual's ability to metabolize and eliminate these pesticides. Changes in paraoxonase I levels may indicate variations in susceptibility to pesticide toxicity. Pesticide exposure has been associated with adverse effects on the hematological system.

 This article brings forth all the required details and has analysed in depth the relation of Paraoxonase I and hemoglobin levels in relation to OPC and Carbamate exposure.

 The structured abstract helps in the understanding of the relevant details of the study. The introduction and methodology is described in detail.The results have been tabulated and significance levels have been discussed. The discussion is elaborate.

 This article has brought forth the importance of  evaluation of paraoxonase I and hemoglobin levels in conjunction with organophosphorus and carbamate levels which provides a comprehensive approach to assessing the health and safety of farmers and agricultural workers exposed to pesticides, offering valuable insights for both individual health management and broader public health considerations.

 I consider this is a remarkable study .

Is the work clearly and accurately presented and does it cite the current literature?

Yes

If applicable, is the statistical analysis and its interpretation appropriate?

Yes

Are all the source data underlying the results available to ensure full reproducibility?

Yes

Is the study design appropriate and is the work technically sound?

Yes

Are the conclusions drawn adequately supported by the results?

Yes

Are sufficient details of methods and analysis provided to allow replication by others?

Yes

Reviewer Expertise:

Forensic Medicine and Toxicology

I confirm that I have read this submission and believe that I have an appropriate level of expertise to confirm that it is of an acceptable scientific standard.

# Reviewer report: Reviewer response for version 2

**DOI:** 10.5256/f1000research.156722.r215720

**Published:** 2024-01-02

**Authors:** Eugenio Vilanova

**Affiliations:** 1 Universidad Miguel Hernandez de Elche, Elche, Valencian Community, Spain

Author have considered my comment and suggestions. Therefore I suggest to APPROVE the manuscript.

Is the work clearly and accurately presented and does it cite the current literature?

Partly

If applicable, is the statistical analysis and its interpretation appropriate?

Partly

Are all the source data underlying the results available to ensure full reproducibility?

Yes

Is the study design appropriate and is the work technically sound?

Partly

Are the conclusions drawn adequately supported by the results?

Partly

Are sufficient details of methods and analysis provided to allow replication by others?

Yes

Reviewer Expertise:

Toxicology of organophosporus compounds

I confirm that I have read this submission and believe that I have an appropriate level of expertise to confirm that it is of an acceptable scientific standard.

# Reviewer report: Reviewer response for version 1

**DOI:** 10.5256/f1000research.144558.r195014

**Published:** 2023-08-22

**Authors:** Eugenio Vilanova

**Affiliations:** 1 Universidad Miguel Hernandez de Elche, Elche, Valencian Community, Spain

In this paper, blood and urine of farmers working 5 years potentially exposed to pesticides were qualitatively detected organophosphorus (OP) and carbamates (CBM) and measured paraoxonase 1 (PON1) and hemoglobin in serum.

 I consider this is an interesting study that does warrant to be published. It is not easy to get direct information about pesticide exposure in humans, therefore any available information is very convenient to be reported, including in studies in which like this, the reported information is very limited.

 However, there are some concerns about the lack of some information, and the interpretation of the observations. Therefore, I suggest authors to consider my comment in order to improve the manuscript.

 Sorry, I apologise that I have done a long series of comments but it reflects my interest in the topic and my willingness to help for improving the manuscript.

 (0). ONE GENERAL CONCERN

 The study is studying 100 people, all them farmers with potential exposure to pesticides. The study is lacking of stabilizing a control group with a clear absence of potential exposure. I understand that author is comparing several groups, and may be considered that the equivalent to a control is the group with negative detection by the qualitative procedures. However, the negative detection by such weak procedure does not means absence of exposure. As I consider that any observed data in humans are convenient to be reported, I still consider of interest its publication beside of this deficiency. In any case authors had to include a short comment about this limitation in the Discussion section.

 (1).-SOME MINOR GENERAL ISSUES:

 (1.1).-As the wording organophosphorus and carbamates are used many times along the manuscript (and more if you consider my next suggestion), I recommend define an abbreviation first time mentioned and use the abbreviation along the manuscript. I suggest to use the most usual abbreviation of OP and CbM or CBM.

 (1.2).-There are several inconsistencies. Note that the word “organophosphorus” is a common name, therefore, it should be named in lower case always (except when initiating a paragraph, or using an abbreviation).

 (1.3).-You are not studying “pesticides” in general, but only OPs and CB, then please delete most of the expression of “pesticides” along the manuscript for the specific kind of evaluated compounds (OP, CB, or OP and CB).

 (1.4).-The number of decimal digits (four) in Table 2 for the values of PON and hemoglobin levels has no meaning as the Standard error are in the level of decimals or units, therefore I suggest to show only a maximum of 2 decimal digits which mean up to 4 total significant digits. The same for the PON1 values in the complementary raw data in the Excel table.

 (2).-A MAIN CONCERN about interpretations:

 In abstract and in several points of the manuscript, it is mentioned that paraoxonase (PON) is altered in worker with positive detection of OP and carbamates. It seems that authors suggest that the change of PON1 is due to the exposure to pesticides, but the interpretation might be just the reverse as I am commenting as follows:

 Paraoxonase is well known that is able to hydrolyze organophosphorus compounds (OPs) (biodegradation and detoxification) but not inhibited by them. Authors could consider the interpretation that just those people with low level of paraoxonase have lower capacity of detoxication, and consequently, higher level may be detected in their blood.

 Actually, for example, in the reference 1, have in the title the same mistake with a inconsistency, as in that paper in the last sentence in abstract, the author concluded with the reverse line of cause-effect relationship: that the level and polymorphism of PON1 is a risk factor affecting to the susceptibility to the exposure to OPs. In your Discussion, I agree with your comment in which you confirm this interpretation. However, at the end of 3rd paragraph you mention this reference (1); I consider that that paper of Sozmen, Mackness et al (2002) did not concluded that OP are directly affecting PON1, although they mentioned this possibility. No confirmation of inhibition of PON1 by OPs have been demonstrated. They actually observed a reduction of level, that could be interpreted by a reduction of liver synthesis.

 So, I strongly suggest author to change the rational interpretation, changing the direction of the correlation PON<> (OP LEVEL AND TOXICITY). PON level is a cause (risk factor), not the effect. PON1 level and polymorphism, is affecting the capacity of detoxication and so the level of OP in blood may be higher as they are hydrolyzed and eliminated at lower rate.

 Therefore, it is not true that OP exposure is strongly altering PON (what is mainly genetically conditioned), while it should be considered that the level of PON1 in the individual is affecting the level of OPs remaining in blood and the possibility of toxic effects.

 This comment does not invalidate the value of the paper and that in my opinion the recorded data is of interest to be published. My concern is with the interpretation.

 (3).-OTHER MINOR ISSUES

 (3.1).-The difference in the levels of paraoxonase and hemoglobin, seems not be statistically significant, therefore author should be more prudent describing the differences in the level of the different groups and indicated in results and abstract expressions as “lower values were observed for OP positive workers, although no statically significant.

 (3.2).-Under my point of view the relationship with PON1 level has actual sense in relationship with OPs. Consequently, it should be of interest comparing all positive OP (group 2+4) with all negative OP (group 1 + 3) cases.

 (3.3).-For hemoglobin, I have more difficult to give an interpretation of why OP positives (group 2 and 4) seems to show lower level . The suggested interpretation of an effects in the cells producing hemoglobin seems a reasonable interesting speculation, although there is not published data supporting it, but perhaps it worth to be investigated in future.

 (4).-ABSTRACT

 (4.1).-Abstract is a very important section of the paper as may reader only read it, and it should reflect clearly and objectively the content of the manuscript. I consider that it needs to be improved significantly.

 (4.2).-In Method in abstract should be mentioned that OP and CB pesticides were detected by a qualitative method with thin layer chromatography, and assigned a positive or negative to OP or carbamates.

 (4.3).-In method in abstract, please indicate the kind of epidemiological study (cross-sectional, longitudinal, other). In title you say “cross sectional”. However, in the abstract you are mentioning 5 years. Have you been studying these workers continually or periodically during this time, or on the contrary, in a specific time but to workers that had been working during the las 5 years?

 (4.4).-Taking into consideration my main concern, please modify abstract to show more objective and precise description of the observations. Some examples as follows:

 The sentence: “The study showed that the pesticides were found in the blood and urine samples of farmers and there was an alteration of paraoxonase I and hemoglobin levels in them due to the exposure of pesticides in large quantities over some time.”

 For something like: “Farmers were classified as a function of positive/negative detection of OP and CB and the level of PON1 and hemoglobin were compared in the different groups.”

 The sentence: “The present study showed around 81% of the participants who were intermittently exposed to pesticides for more than five years were detected with pesticide toxicity.”

 For: “The 81% of the participants showed positive detection for OPs, CB or both”

 (4.5).-You also indicated in abstract that 81% workers “were detected with pesticide toxicity”. What you want to mean with “toxicity”? Are you wanting to say that all they suffer illness or disease and some symptoms and signs of health alteration compatible with toxicity OP/carbamate? In that case, please be more precise and specific, indicating the specific sign of toxicity. I understand that you are not evaluating clinically toxicological effects, therefore delete the wording “toxicity” in all related comments along the manuscript. I understand that you are using as criteria the positive detection with the qualitative methods you have used.

 (4.6) Also, you say that “alteration in the levels of PON1 and hemoglobin in farmers and agricultural workers with **positive organophosphorus and carbamates** in their blood”, however later you say that “done without quantifying the amount of pesticides” Therefore it is no clear how you could consider “positive in OP and CBM in blood? For this reason is needed my suggestion (1.3)

 (5).-please, consider my previous comments before for abstract, also in those place in the section of Methods, Results and Discussion, related with the same topic.

 (6).-INTRODUCTION INITIAL SENTENCE AND LAST SENTENCE IN DISCUSSION

 (6.1).-I disagree and suggest delete or modify your first sentence in Discussion, in which you say: “Pesticides poisoning **is the major global issue** all over the world”. This an absurd exaggeration, compare with many other severe issues in the world (cancer, tobacco, COVID, wars, hunger). Therefore, I suggest to change by something like as: “Pesticide poisoning is an important toxicological issue”.

 The reference (16) says “a major”, while you say “the major”. This reference is not good evidence, because it is just a statement with no information about the studies that demonstrate the figures they show. Therefore may be better to delete as it is not needed for the purpose of you paper.

 (6.2).-Related with the previous comment, I also strongly disagree and I suggest deleting the sentence in the introduction that say “According to World Health Organization (WHO) in 1990, all together around three million pesticide poisoning cases occur annually around the world (WHO 1990)”. This and similar sentences are written in many publications but if you think that it is true, please show an appropriate reference of the original objective study demonstrating such statement. Please do not give an indirect reference of reference of reference. It is also common to state that 300.000 fatalities due to OPs intoxication is occurring every year. There are some reports (not official from WHO but supported by WHO) in which, based of some few data were suggesting such kind of figures. These statements are coming from reports in 1990, from data coming from 1970s, without appropriate sufficient data obtained from regions that are not representing the world. It has been assumed that data from a few situations with special incidence due to special social circumstances, can be extrapolated to all the world, and that today is still occurring the same situation. In those statements, 3 million of cases are also suggested from which 2 million by suicides and the other 1 M by occupational and other unintentional intoxication. In India it was officially recorded about 400,000 suicides during 11 years (1995-2015) by pesticides (around 35,000 `per year) with about 20% fatalities, 90 % by OPs and with a strong tendency to decrease after 2014. If in India with not very good situation with 1.4 thousand million inhabitants, assuming all the world with 8 thousand million people in the same bad situation than India, the extrapolation would be about 300,000 cases (very far from the 2 million in those reports). Assuming about 20% fatalities as described in India, they would be not more than 60.000. Indeed, this extrapolated number would be a very high exaggeration, because the tendency since 2010s is a clear decrease, OPs is strongly decreasing their use in all the world. For example, in my country (40 million population) zero lethal cases by pesticides have been registered during last 5 years.

 Can you suggest an original objective study demonstrating that 3 million pesticide acute intoxication? Probably not, therefore it may be better to delete it to avoid something not sure and discussable, and what is not needed for the purpose of your paper. Probably a more simple and neutral statement as “Exposure to pesticides and specially of OPs is an important toxicological issue of concern, in India” This problem is still a big problem in some countries, and may be better to give reference about the specific situation in your country with objective data. If you have not such appropriate reference, better not to give not validated data. You do not need for the purpose of your paper that I still consider of interest its publication.

Is the work clearly and accurately presented and does it cite the current literature?

Partly

If applicable, is the statistical analysis and its interpretation appropriate?

Partly

Are all the source data underlying the results available to ensure full reproducibility?

Yes

Is the study design appropriate and is the work technically sound?

Partly

Are the conclusions drawn adequately supported by the results?

Partly

Are sufficient details of methods and analysis provided to allow replication by others?

Yes

Reviewer Expertise:

Toxicology of organophosporus compounds

I confirm that I have read this submission and believe that I have an appropriate level of expertise to confirm that it is of an acceptable scientific standard, however I have significant reservations, as outlined above.

# Response

**Published:** 2023-09-27

**Authors:** Shankar Bakkannavar

**Affiliations:** Kasturba Medical College Hospital, India

Dear Reviewer,

 Thanks a lot for detailed review and letting us know the points to be added/modified in our manuscript. Accordingly we have modified the manuscript pointwise as per your suggestions which are attached below for your perusal.

1. The study is lacking of stabilizing a control group with a clear absence of potential exposure. I understand that author is comparing several groups, and may be considered that the equivalent to a control is the group with negative detection by the qualitative procedures.

In our study the control group was comprised of participants who were non farmers. The estimation of samples did not reveal the presence of OP and CBM in these samples.

1. I suggest to use the most usual abbreviation of OP and CbM or CBM.

Changed

1. You are not studying “pesticides” in general, but only OPs and CB, then please delete most of the expression of “pesticides” along the manuscript for the specific kind of evaluated compounds (OP, CB, or OP and CB)

Changes done. However at few places we have kept pesticides as we are introducing the topic and wherever necessary we have removed pesticides and and change to organophosphorus and carbamates.

1. Table 2 for the values of PON and hemoglobin levels has no meaning as the Standard error are in the level of decimals or units, therefore I suggest to show only a maximum of 2 decimal digits which mean up to 4 total significant digits. The same for the PON1 values in the complementary raw data in the Excel table.

Changes done and only 2 decimal points included.

1. ON<> (OP LEVEL AND TOXICITY). PON level is a cause (risk factor), not the effect. PON1 level and polymorphism, is affecting the capacity of detoxication and so the level of OP in blood may be higher as they are hydrolyzed and eliminated at lower rate.

Your statement if highly appreciated and we have added the same thing in the discussion part of our manuscript.

1. Therefore, it is not true that OP exposure is strongly altering PON (what is mainly genetically conditioned), while it should be considered that the level of PON1 in the individual is affecting the level of OPs remaining in blood and the possibility of toxic effects.

**PON level is a risk factor, not the effect. Its level and polymorphism is affecting the capacity of detoxication and so the level of OP in blood may be higher as they are hydrolysed and eliminated at lower rate. Therefore, it should be considered that the level of PON1 in the individual is affecting the level of OPs remaining in blood and the possibility of toxic effects.**

1. The difference in the levels of paraoxonase and hemoglobin, seems not be statistically significant, therefore author should be more prudent describing the differences in the level of the different groups and indicated in results and abstract expressions as “lower values were observed for OP positive workers, although no statically significant.

When the difference in the levels of paraoxonase and hemoglobin was considered, the lower values were observed for OP and CBM positive workers, although it was not statically significant.

1. Under my point of view the relationship with PON1 level has actual sense in relationship with OPs. Consequently, it should be of interest comparing all positive OP (group 2+4) with all negative OP (group 1 + 3) cases.

Values were compared between groups 2 & 4.

**Group**

**N**

**Mean**

**Median**

**SD**

**SE**

**df**

**P**

 Paroxanase

 2

 13

 29.3

 29.3

 0.976

 0.271

 19

 <0.001

 4

 8

 35.6

 35.5

 1.16

 0.411

1. Abstract is a very important section of the paper as may reader only read it, and it should reflect clearly and objectively the content of the manuscript. I consider that it needs to be improved significantly.

It is improved by changing the statements as we have a word limit to structure the abstract.

1. In Method in abstract should be mentioned that OP and CB pesticides were detected by a qualitative method with thin layer chromatography and assigned a positive or negative to OP or carbamates.

Included the suggested point in the manuscript.

1. In method in abstract, please indicate the kind of epidemiological study (cross-sectional, longitudinal, other). In title you say “cross sectional”. However, in the abstract you are mentioning 5 years. Have you been studying these workers continually or periodically during this time, or on the contrary, in a specific time but to workers that had been working during the las 5 years?

Cross sectional study was included.

1. “The study showed that the pesticides were found in the blood and urine samples of farmers and there was an alteration of paraoxonase I and hemoglobin levels in them due to the exposure of pesticides in large quantities over some time.”

Changes done in the manuscript.

1. Farmers were classified as a function of positive/negative detection of OP and CB and the level of PON1 and hemoglobin were compared in the different groups.

Values were compared between groups 1 & 3 and 2 & 4.

1. The present study showed around 81% of the participants who were intermittently exposed to pesticides for more than five years were detected with pesticide toxicity.

Changes done

1. The 81% of the participants showed positive detection for OPs, CB or both

Changes done

1. delete the wording “toxicity” in all related comments along the manuscript.

Toxicity word deleted wherever it was appropriate.

1. in their blood”, however later you say that “done without quantifying the amount of pesticides” Therefore it is no clear how you could consider “positive in OP and CBM in blood?

Positive or negative was decided by qualitative method (TLC).

1. “Pesticides poisoning is the major global issue all over the world”. This an absurd exaggeration, compare with many other severe issues in the world (cancer, tobacco, COVID, wars, hunger). Therefore, I suggest to change by something like as: “Pesticide poisoning is an important toxicological issue”.

This Statement is considered and added

1. The reference (16) says “a major”, while you say “the major”.

Reference 16 is deleted as suggested.

1. “Exposure to pesticides and specially of OPs is an important toxicological issue of concern, in India”

Statement was considered in the manuscript.
